# Editorial: [Application of stem cells in the treatment of myocardial infarction]

**DOI:** 10.3389/fcvm.2023.1333732

**Published:** 2023-11-29

**Authors:** Cheuk-Kwan Sun

**Affiliations:** ^1^ Department of Emergency Medicine, E-Da Dachang Hospital, I-Shou University, Kaohsiung, Taiwan; ^2^ School of Medicine for International Students, College of Medicine, I-Shou University, Kaohsiung, Taiwan

**Keywords:** ischemic heart disease, mesenchymal stem cells, induced pluripotent stem cells, ischemia-reperfusion injury, cardiovascular tissue engineering

**Editorial on the Research Topic** [Application of stem cells in the treatment of myocardial infarction]

Myocardial infarction remains one of the top killers in the developed world. In contrast to conventional coronary intervention in the treatment of ischemic heart disease, the use of stem cells has unleashed the therapeutic potential of a novel biological approach. Taking into account the similarity in cardiac anatomy and function between pigs and humans, a preclinical study using a porcine balloon-induced myocardial infarction model by Chen et al. with a follow-up period up to five months has demonstrated a significantly larger reduction in infarct size as well as better preservation of left ventricular function in animals having received left intracoronary arterial administration of human bone marrow-derived mesenchymal stem cells 90 min after infarct induction compared to those without stem cell treatment, thereby paving the way for further clinical trials.

Moreover, given that the goal of cardiovascular tissue engineering is to establish functional scaffolds with biologically compatible materials to provide a cellular environment for restoring the functions of damaged tissues or organs, the therapeutic potential of stem cells against myocardial infarction and ischemic cardiomyopathy has triggered the development in this discipline. A bibliometric analysis of 2,273 documents in the field of cardiovascular tissue engineering published in recent three decades conducted by Lai et al. identified functional maturation of stem cells and the application of biomaterials as potential hotspots in this research area, further highlighting the mutual positive influences between tissue engineering development and the clinical application of stem cells.

Besides, the clinical applicability of stem cell therapy is underscored by its ubiquity. In addition to common sources such as adipose tissue, blood, and bone marrow, induced pluripotent stem cells (iPSCs) generated directly from somatic cells have sparked a keen interest in research because of the promising implication of an unlimited supply of stem cells. Nevertheless, there have been concerns over the safety of iPSCs due to their malignant potential. To address this issue, a human clinical trial examined the feasibility of allogeneic induced pluripotent stem cell (iPSC)-derived cardiomyocyte patch in the treatment of ischemic cardiomyopathy. That study by Kawamura et al., which reported three patients with severely compromised cardiac function, revealed an improvement in heart failure symptoms without accompanying transplanted cell-related adverse events after one-year follow-up. Intriguingly, the authors also noted improved left ventricular contractility as well as myocardial blood flow in the three patients following three-month immunosuppressant administration. The results, therefore, support the safety of iPSC use in patients with severely compromised heart functions although further clinical studies are warranted to reinforce their findings.

Another question regarding the use of iPSCs is their fate after administration. Utilizing an immunodeficient rat model, Saito et al. attempted to test the feasibility of non-invasive *in vivo* tracking of transplanted cardiomyocytes derived from human iPSCs using the technique of nuclear medicine imaging. Human iPCSs, after being transduced with sodium/iodide symporter (NIS), were subcutaneously implanted into the animals. The resulting teratomas were successfully detected with single photon emission computed tomography (SPECT/CT) after six weeks, thereby reinforcing the applicability of this technique in tracing the implanted stem cells. Nevertheless, further studies are required to assess the effect of symporter transduction on stem cell function as well as the safety of radioactive tracer use.

**Figure 1:**
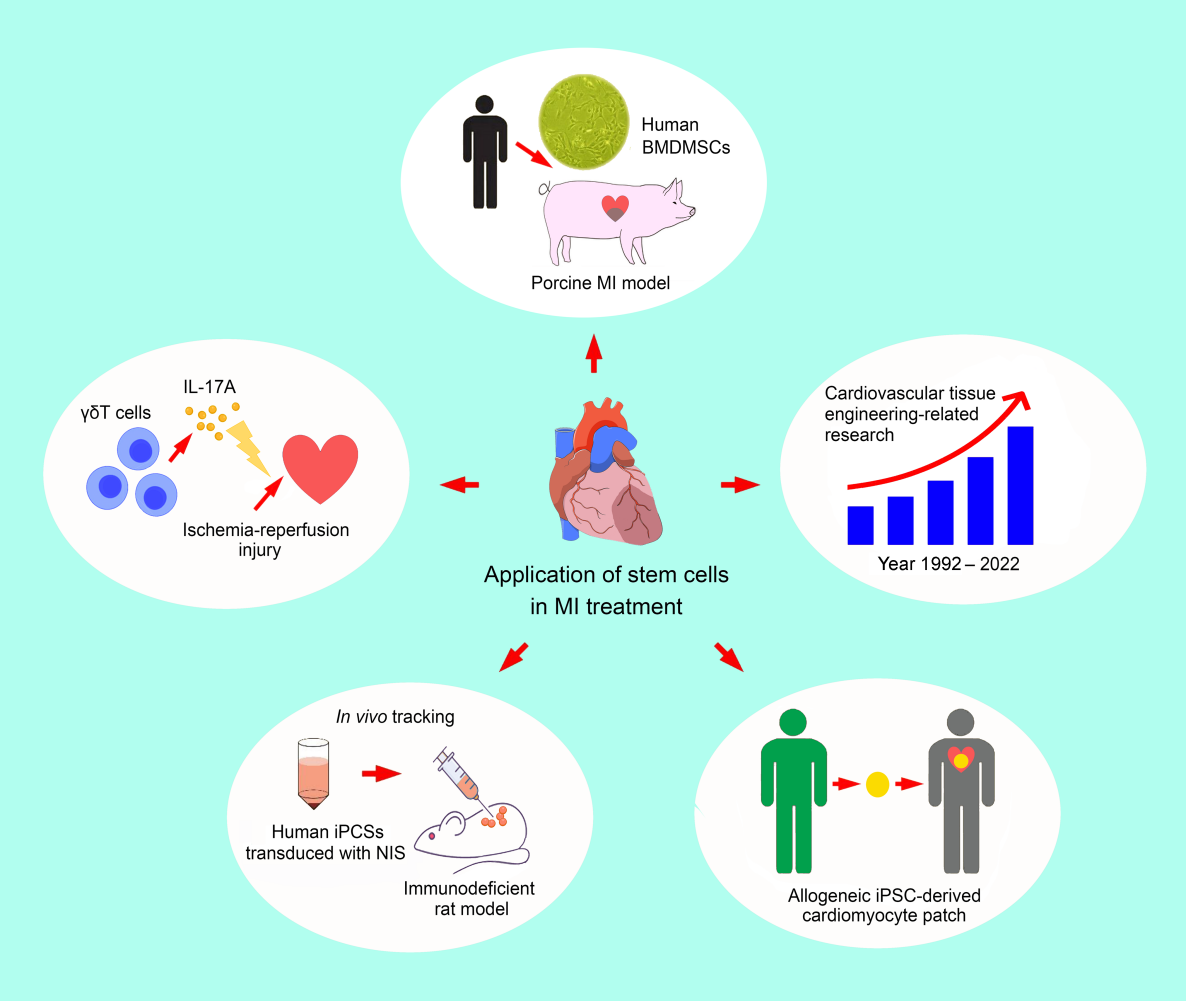
An overview of the five studies included in the current special issue, highlighting the increasing interest in and diversity of cellular therapeutic strategies against ischemic heart disease. BMDMSCs: bone marrow-derived mesenchymal stem cells; iPCS: induced pluripotent stem cell; IL-17A: interleukin-17A; MI: myocardial infarction; NIS: sodium/iodide symporter.

One further step that needs to be taken to complete the jigsaw puzzle is a deeper understanding of the biological mechanisms underpinning the observed stem cell therapeutic efficacy. Ischemia-reperfusion injury, the severity of which is proportional to the duration of ischemia of an organ followed by a restoration of blood flow, is known to involve the activation and release of inflammatory mediators and is common in patients experiencing myocardial infarction. In addition to the ischemic injuries from the initial myocardial infarction, ischemia-reperfusion contributes to further myocardial damage after resuming the patency of the obstructed coronary artery. To elucidate the underlying immunological mechanism, Luo et al. investigated the role of γδT cells (i.e., one of the two main groups of T cells) in the process. The authors reported that IL-17A, which is the core cytokine of γδT-mediated immune response, could be protective against cardiac ischemia-reperfusion insult. The finding is of potential clinical interest as it bears significant immunological implications that may guide the direction of the development of appropriate stem cell strategies for treating myocardial infarction as well as the subsequent ischemia-reperfusion injuries that adversely impact the prognosis of this patient population. The intricate immunological network underlying cellular protection against myocardial ischemic diseases warrants further explorations. Figure 1 is an overview of the five studies included in this special issue.

In conclusion, as evidence accumulates regarding the biomolecular mechanisms involved in the therapeutic effects of stem cells against ischemic heart disease, it is foreseeable that stem cell therapy will have a significant part to play in the treatment of myocardial infarction either alone or in combination with other interventional strategies. Notwithstanding the apparently encouraging outcomes from previous studies, further large-scale clinical trials are needed to consolidate the role of stem cell therapy as a routine evidence-based clinical practice.

